# Low-Dose of Bergamot-Derived Polyphenolic Fraction (BPF) Did Not Improve Metabolic Parameters in Second Generation Antipsychotics-Treated Patients: Results from a 60-days Open-Label Study

**DOI:** 10.3389/fphar.2017.00197

**Published:** 2017-04-11

**Authors:** Antonio Bruno, Gianluca Pandolfo, Manuela Crucitti, Massimo Cacciola, Vincenza Santoro, Edoardo Spina, Rocco A. Zoccali, Maria R. A. Muscatello

**Affiliations:** ^1^Department of Biomedical and Dental Sciences and Morphofunctional Imaging, University of MessinaMessina, Italy; ^2^Department of Clinical and Experimental Medicine, University of MessinaMessina, Italy

**Keywords:** bergamot-derived polyphenolic fraction, metabolic syndrome, body weight, hyperlypemia, hyperglycemia, second generation antipsychotics

## Abstract

**Objectives:** The nutraceutical approach to the management of metabolic syndrome (MetS) might be a promising strategy in the prevention of cardio-metabolic risk. Low-dose bergamot-derived polyphenolic fraction (BPF) has been proven effective in patients with MetS, as demonstrated by a concomitant improvement in lipemic and glycemic profiles. The present study was aimed to further explore, in a sample of subjects receiving second generation antipsychotics (SGAs), the effects on body weight and metabolic parameters of a low dose of BPF (500 mg/day) administered for 60 days.

**Methods:** Twenty-eight outpatients treated with SGAs assumed BPF at single daily dose of 500 mg/day for 60 days. Body weight, BMI, fasting levels of glucose, total cholesterol, high density lipoprotein cholesterol, low density lipoprotein cholesterol and triglycerides were determined; moreover, Brief Psychiatric Rating Scale (BPRS) was administered.

**Results:** Low-dose BPF administration did not change clinical and metabolic parameters, as well as clinical symptoms in the study sample. At the end of the trial, among completers (*n* = 24) only nine patients (37.5%) reached an LDL reduction >0 but <50%.

**Conclusions:** Our results demonstrate that patients treated with SGAs may need higher BPF doses for obtaining the positive effects on body weight and metabolic parameters previously found in the general population at lower doses.

## Introduction

Metabolic syndrome (MetS), a cluster of risk factors including obesity, dyslipidemia, glucose intolerance, insulin resistance (or hyperinsulinemia) and hypertension, is highly predictive of type 2 diabetes and cardiovascular disease (Grundy et al., 2004).

People with psychiatric disorders usually show a high incidence of MetS, and especially schizophrenia subjects exhibit prevalence rates much higher than general population and healthy controls (Casey, 2005). Although schizophrenia appears to be able to represent itself a risk factor for MetS, such risk is further exacerbated by the use of atypical antipsychotic agents (Haddad, 2004; Coccurello et al., 2009; Coccurello and Moles, 2010a,b; Miron et al., 2014). Moreover, atypical antipsychotics polytherapy, compared to monotherapy, may be independently associated with an increased risk of pre-MetS and with high rates of MetS (Reynolds and Kirk, 2010; Misawa et al., 2011).

Despite the availability of multiple pharmacological interventions for the treatment of MetS-related alterations, these therapeutic strategies often present several side effects, such as myalgia, myopathy, liver disease and rhabdomyolysis and, furthermore, an inadequate control of cardiovascular disease (Alsheikh-Ali and Karas, 2009; Joy and Hegele, 2009), thus suggesting the need to find new potential therapies.

The nutraceutical approach to the management of MetS might be a promising strategy in the prevention of cardio-metabolic risks. In particular, the polyphenols used in clinical practice have demonstrated to be effective in the treatment of diabetes mellitus, MetS and their complications and to be able to positively modulate multiple biochemical and clinical end-points (Davì et al., 2010).

Bergamot (Citrus bergamia) is an endemic plant of the Southern Italy, which differs from other citrus fruits for flavonoids and flavonoid glycosides composition (neoeriocitrin, neohesperidin, naringin, rutin, poncirin, roifolin, and neodesmin), and for their high amount (Nogata et al., 2006; Misawa et al., 2011). As well as to a non-specific antioxidant action, lipid-lowering and glucose-lowering actions of BPF may be attributed to several specific mechanisms (Fraga et al., 2010). In particular, the hypolipidemic effect is related to the selective inhibition of the hydroxymethylglutaryl CoA reductase and to the reduction of the activity of phosphatidate phosphohydrolase enzymes, whereas the hypoglycemic action is associated with the increase of the activities of AMP kinase, sirtuin-1 and, in muscle cells and liver, glucose transporter GLUT-4 (Ciccone et al., 2015).

Recently, the therapeutic potential of bergamot derivatives has been investigated in human studies: it was shown that bergamot-derived polyphenolic fraction (BPF) has beneficial effects in patients with MetS, as demonstrated by a concomitant improvement in lipemic and glycemic profiles, and by the improvement of altered endothelium-mediated vasodilation (Mollace et al., 2011; Gliozzi et al., 2014; Toth et al., 2016). Specifically, Mollace et al. (2011) have demonstrated that BPF was effective already after the administration of 500 mg/day for 30 days, and that differences between 500 and 1,000 mg dosages were statistically significant only for high density lipoprotein (HDL) cholesterol. More recently, we tested the efficacy of BPF at the daily dose of 1,000 mg administered for 30 days in a sample of 15 outpatients treated with second generation antipsychotics (SGAs); our findings were encouraging, since BPF administration resulted in a statistically significant reduction of body weight (*P* = 0.004) and in a trend for body mass index (BMI) decrease (*P* = 0.005); however, no effects on metabolic parameters were observed (Bruno et al., 2016).

Based on the evidence from the literature, including our previous clinical results, the present study was aimed to further explore, in a sample of subjects receiving SGAs, the effects on body weight and metabolic parameters of a low dose of BPF (500 mg/day) administered for 60 days.

## Materials and methods

### Study design

This was a 60-day, open-label, preliminary study aimed to evaluate the efficacy and safety of adjunctive BPF to atypical antipsychotics therapy. BPF (Bergamot Polyphenolic Fraction BPF®, H&AD S.r.l—Bianco (RC), Italy) was administered in capsules at the oral daily dose of 500 mg/day and was maintained unchanged until the end of the trial at day 60. The concentration of 5 main flavonoids per capsule was: neoheriocitrin (55,535 mg), naringin (58,903 mg), neohesperidin (62,966 mg), melitidin (7,958 mg) and brutieridin (24,371 mg); excipients of any type were not present. During the study, no additional medications, including aspirin or non-steroidal anti-inflammatory drugs, were allowed. The study was carried out at the Psychiatry Unit of the University Hospital of Messina, Italy. The protocol has been approved by the Ethics Committee of the University of Messina, Italy.

### Subjects

Twenty-eight outpatients, 15 men and 13 women, aged 21–65 years, in treatment with SGAs (clozapine, olanzapine, quetiapine, risperidone and paliperidone), were included in this study. All patients had been on monotherapy for at least 3 months; the dose had been stable for at least 1 month before the study and was left unchanged throughout the study. The patients did not receive any antidepressant or anticonvulsant drugs for a period of 2 months before the study. Furthermore at enrollment, all subjects received standardized dietary advice in order to reduce the variability of their baseline metabolic values. Patients with any significant concurrent medical illnesses, organic brain disorder, mental retardation, pregnant or lactating women, or a current diagnosis of alcohol/drug dependence were excluded. All the patients provided written informed consent after a full explanation of the protocol design, and the study was conducted according to the Declaration of Helsinki.

### Assessment

Patients attended three visits: initial screening (week −1), inclusion (day 0), and final visit (day 60).

A physical examination was performed to measure vital signs [blood pressure, heart rate, body weight (BW) and body mass index (BMI)].

Standard laboratory methods were used to determine fasting levels of glucose, total cholesterol, HDL, and triglycerides. Low density lipoprotein (LDL) cholesterol was determined by the Friedewald et al. calculation (LDL = total cholesterol – (HDL + [triglycerides/5]) (Friedewald et al., 1972).

The Brief Psychiatric Rating Scale (BPRS) (Overall and Gorham, 1962) was administered by two senior psychiatrists with at least 5 years of clinical experience and trained on rating scales; each patient had the same person conducting clinical interviews and administering psychometric and neuropsychological tests.

All physical information, laboratory measurements and data for clinical and neuropsychological assessments were obtained at baseline and at the end of the study (approximately 60 days apart).

Adverse effects, either observed or spontaneously reported, were recorded at each visit and classified in terms of onset, duration, severity, action taken, and outcome. In order to monitor the adherence to the study protocol, weekly telephone calls during the study period and a pill count on the last day (day 60) were carried out.

### Measurement of serum concentrations of antipsychotics

Steady-state serum concentrations of clozapine, olanzapine, risperidone, 9-hydroxyrisperidone (or paliperidone) and quetiapine were measured by HPLC at day 0 and day 60 (Avenoso et al., 1998, 2000; D'Arrigo et al., 2006; Santoro et al., 2008).

### Statistical analysis

Before the start of the study, sample size was calculated: under the assumption of a significant level of 0.05 with a power of 0.80 and an effect size of 0.50, a minimal sample size of 28 subjects was determined. Due to the small sample size, the analyses were carried out by non-parametric tests. An intention-to-treat analysis (ITT) with last-observation-carried forward (LOCF) was performed. Continuous data were expressed as mean ± SD and the within group differences in efficacy ratings between baseline and final test were analyzed by the Wilcoxon rank sum test. To measure the magnitude of a treatment effect, effect size was provided by using Cohen's d statistic and was considered small when lower than 0.50, moderate when ranging from 0.50 to 0.79, and large when equal to or greater than 0.80. The statistical analysis was performed with SPSS 16.0 software (SPSS Inc., Chicago, Ill).

## Results

Baseline characteristics, duration of SGAs prior treatment, SGAs type and daily dose in enrolled/completers subjects are detailed in Table 1. Twenty-four patients completed the study (85.7% completion rate); there were four premature dropouts, due to non-compliance with the visits.

**Table 1:** **Demographic and clinical characteristics of the subjects enrolled**.

Patients entered/completers	28/24
Sex (M/F)	15/13
Age (years), mean ± SD	45.8 ± 11.7
Educational level (years), mean ± SD	9.6 ± 3.1
Duration of prior AP treatment (months), mean ± SD	25.6 ± 34.1
**SGAs (Dose range – mg/d)**	**n. entered/completers**
Olanzapine (5–20 mg/d)	7/7
Clozapine (100–350 mg/d)	7/6
Quetiapine (50–100 mg/d)	6/5
Risperidone (3–4 mg/d)	4/3
Paliperidone (9–12 mg/d)	4/3

### Treatment response

Table 2 shows the baseline and final values of the different vital signs (blood pressure, heart rate, BW and BMI) and metabolic parameters (triglycerides, total cholesterol, HDL, LDL, and glucose), and the effect size for the sample group: at day 60, within-group comparison revealed that BPF administration did not change the values of the considered parameters. Effect sizes were small in each explored clinical and metabolic parameters.

**Table 2:** **Clinical and metabolic changes, and effect sizes for efficacy measures in patients receiving BPF at baseline and day 60 (LOCF)**.

	**Baseline (T0)**		**Day 60 (T1)**		**Wilcoxon test T0 vs. T1**		**Cohen's d**
	**Mean**	**SD**	**Mean**	**SD**	***Z***	***P***	
Systolic blood pressure (mm HG)	122.20	17.26	124.96	14.96	−1.053	0.292	0.2
Diastolic blood pressure (mm HG)	75.88	9.40	77.56	9.04	−0.888	0.375	0.2
Heart rate (b/m)	80.92	12.93	82.92	16.46	−0.713	0.476	0.1
Body weight (kg)	84.07	23.18	84.20	23.54	−0.190	0.849	0
Body mass index (kg/m^2^)	30.50	7.55	30.52	7.57	−0.047	0.962	0
Triglycerides (mg/dl)	109.32	51.94	120.56	64.76	−1.141	0.254	0.2
Total cholesterol (mg/dl)	202.56	32.97	205.84	23.80	−1.330	0.184	0.1
HDL (mg/dl)	55.92	19.58	56.21	17.20	−0.244	0.807	0
LDL (mg/dl)	124.77	26.85	125.52	20.13	−0.357	0.721	0
Glucose (mg/dl)	93.56	17.4	95.76	20.82	−1.596	0.111	0.1

*LOCF indicates last observation carried forward*.

### Response rate

LDL reduction was chosen for treatment efficacy evaluation because it is the most commonly used surrogate endpoint for cardiovascular disease reduction, and it is the primary target for statin therapy in cholesterol-lowering guidelines (Perk et al., 2012). Treatment response was defined as a reduction in LDL >0 but <50% or ≥50%, according to the current statin guidelines (Perk et al., 2012; Stone et al., 2014).

Among completers (*n* = 24), 9 individuals (37.5%) experienced an LDL reduction >0 but <50%, whereas 15 subjects (63.5%) experienced no reduction or an increase in LDL compared with baseline. None of the patients experienced an LDL reduction ≥50%.

### Adverse effects

BPF adjunctive treatment was well tolerated at 500 mg/day dosage: no adverse effects were recorded in our sample during the 60-days treatment period. Moreover, regarding clinical symptoms, no significant changes emerged at the end of the study in the BPRS scores (Table 3), suggesting that psychopathological symptoms did not worsen during the trial. Finally, no acute extrapyramidal effects, seizures, or cardiac events occurred.

**Table 3:** **Psychopathological changes, and effect sizes for efficacy measures in patients receiving BPF at baseline and day 60 (LOCF)**.

	**Baseline (T0)**		**Day 60 (T1)**		**Wilcoxon test T0 vs. T1**		**Cohen's d**
	**Mean**	**SD**	**Mean**	**SD**	**Z**	***p***	
**BPRS**							
Anxiety-depression	8.80	3.66	8.88	3.48	0.000	1.000	0
Anergia	7.76	3.52	7.56	3.48	−0.603	0.546	0
Thinking disorder	6.12	2.22	5.72	2.52	−1.411	0.158	0.2
Activity	5.56	1.89	5.52	1.91	−0.104	0.917	0
Hostility-suspicion	4.92	2.73	4.68	2.19	−0.256	0.798	0.1
Total score	33.16	7.90	32.36	8.95	−0.706	0.480	0.1

*LOCF indicates last observation carried forward*.

### Serum concentrations of antipsychotics

Serum concentrations of clozapine, olanzapine, risperidone, 9-hydroxyrisperidone (or paliperidone) and quetiapine were not affected significantly by administration of BPF. Concerning the two antipsychotics more frequently administered to patients participating to the study, namely olanzapine (7 patients) and clozapine (6 patients), mean steady-state serum concentrations of olanzapine were 29.1 ± 12.4 ng/mL at day 0 and 25.0 ± 9.7 ng/mL at day 60, while serum concentrations of clozapine were 551 ± 139 ng/mL at day 0 and 572 ± 124 ng/mL at day 60.

## Discussion

The metabolic effects of atypical antipsychotics represent a serious medical problem to be addressed with different therapeutic approaches in order to improve the life expectancy of subjects with mental disorders. Cross-sectional studies have reported MetS prevalence rates of 11–69% in pharmacologically treated psychiatric patients, and of 4–26% in drug-naïve patients; longitudinal studies have showed baseline prevalence rates between 0 and 14% in drug-naïve patients, increased up to a maximum of 52% after 3 months of antipsychotics treatment (Malhotra et al., 2013). Among SGAs, clozapine, olanzapine, and quetiapine are particularly associated with weight gain, as well as with adiposity-dependent and possibly adiposity-independent glucose dysregulation and dyslipidemia (Newcomer, 2007).

In a previous study, we found that a moderate BPF dose (1,000 mg) for 30 days significantly reduced body weight in SGAs-treated patients, suggesting that BPF could be an effective and safe agent to prevent weight gain associated with atypical antipsychotic use (Bruno et al., 2016). On the basis of such encouraging results, we had the aim to verify the potential use of low-dose BPF administered for a longer period.

Our findings indicated that adjunctive BPF treatment at the oral dose of 500 mg/day administered for 60 days appeared to be scarcely effective for reducing vital signs and metabolic parameters in the study sample. At the end of the trial only nine patients (37.5%) reached a minimal LDL reduction according to the current statin guidelines (Perk et al., 2012; Stone et al., 2014), whereas no patients experienced an LDL reduction ≥50%.

BPF adjunctive treatment was well tolerated at 500 mg/day dosage, as shown by the lack of pharmacokinetic effects on hematic concentrations of antipsychotics, and by the dropout causes, all unrelated to adverse and/or unwanted events, confirming previous studies on safety profile of bergamot extract (Mollace et al., 2011; Gliozzi et al., 2014; Toth et al., 2016). Moreover, regarding psychopathological symptoms, BPF add-on treatment did not modified the explored domains, since no significant changes in BPRS scores were observed.

Our previous results (Bruno et al., 2016) showing a significant reduction of body weight with BPF (1,000 mg/day) supplementation were not confirmed at lower doses; furthermore, our findings did not confirm the beneficial effect of low-dose BPF on lipid and glycemic profiles previously found in the literature (Mollace et al., 2011). In the attempt to explain such results, it should be borne in mind that the pharmacological mechanisms underlying the association of antipsychotics with metabolic disorders (Taylor and McAskill, 2000; Matsui-Sakata et al., 2005) may be different from the mechanisms responsible for MetS in patients who do not take this class of drugs.

The findings of the present study need to be carefully interpreted because of the presence of several limitations, such as the small sample size, the open design, and the lack of a control group. Additionally, all participants in this study belonged to a restricted area of Southern Italy; due to differences in genes, lifestyles and diets (e.g., Mediterranean diet) among ethnic groups, it seems difficult to generalize these results to other populations.

Our current and earlier results tend to suggest that patients treated with SGAs may need higher BPF doses (>1,000 mg) for obtaining the positive effects on body weight and metabolic parameters previously found in the general population at lower doses.

Further clinical trials with adequately-powered and well-designed methodology are needed to better evaluate the effectiveness of the BFP on SGA-induced metabolic side effects, and to identify potential nutraceutical strategies for the prevention of MetS in subjects with mental disorders.

## Author contributions

AB, RZ, and MM designed the study, wrote the protocol, and supervised the Method procedures, the various drafts and the final version of the manuscript. GP and ES managed the literature searches and wrote the first drafts of the manuscript. MCr, MCa, and VS organized recruitment and collected data, contributed to the literature search and writing of the manuscript. All Authors contributed to and have approved the final manuscript.

### Conflict of interest statement

ES has previously received honoraria for speaking and consultation from AstraZeneca, Boheringer-Ingelheim, Eli Lilly, Janssen, Lundbeck, and Pfizer. The remaining authors declare no conflict of interest.
